# Morpho-Physiological Responses of Selected Vegetables in Hydroponic and Soil-Based Systems Under Climatic Stress

**DOI:** 10.21315/tlsr2025.36.3.8

**Published:** 2025-10-31

**Authors:** Syeda Zoia Ali Zaidi, Shaheen Begum, Mehwish Jamil Noor, Gul-e-Saba Chaudhry, Shahbaz Khan, Muhammad Adnan

**Affiliations:** 1 Environmental Sciences Department, Fatima Jinnah Women University, Rawalpindi, Kachari Chowk 46000, Pakistan; 2 Institute of Climate Adaptation and Marine Biotechnology, Universiti Malaysia Terengganu, 21030 Kuala Nerus, Terengganu, Malaysia; 3 Department of Robotics and AI, School of Mechanical and Manufacturing Engineering, National University of Sciences and Technology (NUST), Islamabad, Pakistan; 4 Department of Botany, Kohat University of Science and Technology, Kohat-26000, Pakistan

**Keywords:** Climate Change, Food Insecurity, Hydroponic System, Morpho-Physiological Response, Soil Nutrient Analysis, Crop Yield Improvement

## Abstract

An extreme climatic change due to anthropogenic activities causes disruptions in ecosystems and threatens the planet’s overall balance. Hydroponic is smart and sustainable agriculture practice that aims to produce two times more yield than traditional practices. To investigate the efficiency of hydroponics technique, the morpho-physiological responses of selected vegetable species were analysed. Tomato (*Solanum lycopersicum* L.), Eggplant (*Solanum melongena*), Lettuce (*Lactuca sativa*), Green Chili (*Capsicum annuum*) and Okra (*Abelmoschus esculentus*) were selected for the experiment. Soil nutrients analysis and hydroponics nutrients uptake analysis were also carried out side by side using UV-Visible Spectroscopy, Atomic Absorption Spectroscopy and Titration method. In hydroponic water analysis, it was found that 42% of supplied Cl− had been taken up by the plants whereas 79% of all supplied Zinc and Iron had been taken up by the plants. The uptake percentages of other anions and cations ranged between 45% to 62%. Morpho-physiological responses of Lettuce and Tomato in soil-based and hydroponic experiments were almost similar. Whereas, hydroponically grown Okra, Green Chili and Eggplant showed maximum height, roots length, number of leaves and weight. Overall findings showed that hydroponic system was more efficient in terms of crops yield, water usage and environmental contamination. Thus, it is recommended to increase the duration of experiment in future to further verify the climatic change effects.

**HIGHLIGHTS**

- Hydroponics enhanced nutrient-use efficiency with superior Zn, Fe and Cl^−^ uptake.
- Significant improvement in morpho-physiological traits observed in Okra, Chili and Eggplant.
- Hydroponics maximised yield, resource-use efficiency and minimised environmental footprint.

## INTRODUCTION

Agriculture as a multidisciplinary approach encompasses both arts and science, involves the cultivation of crops and growing livestock. Despite advancements in agricultural technology, traditional farming methods persist globally, exacerbating soil degradation. Pakistan is one of the developing countries and the agricultural sector is considered as its economy’s backbone. To meet the food demand of this increasing population under climatic stresses, rapid urbanisation and deforestation; farmers are under great burden to produce maximum quantities of crops at a faster rate. For this, farmers use different types of chemical fertilisers and pesticides extensively to enhance crop yields and protect them from pests’ attacks. This use of fertiliser/pesticides is affecting soil fertility too (Khan *et al*. 2018). Climatic changes such as sudden fluctuations in rainfall, humidity, temperature and weather patterns pose a great effect on many crops of Pakistan, such as wheat, maize, corn rice, mint saffron, etc. (Alsamir *et al*. 2017). Furthermore, ethnobotanical surveys conducted in Pakistan have documented that several plant species function dually as food and sources of therapeutic agents within indigenous healthcare systems (Adnan *et al*. 2014; Tariq, Mussarat, *et al*. 2015; Tariq, Adnan, *et al*. 2016). This multifunctionality underscores the significance when evaluating alternative cultivation approaches, such as hydroponic production under climatic stress conditions. Hydroponic system or hydroponics is one of the advanced sustainable agricultural techniques that produces 2 to 3 times higher yield and eliminates the use of other inert mediums like soil, sand and gravel, thus protecting soil from contamination and infertility. Hydroponics is the type of hydroculture in which crops or a plant grows entirely in water. This system can grow any type of plant as the roots of plants are completely exposed to nutrient rich solutions containing all the essential micro and macronutrients taken up by plants through soil (Son *et al*. 2020). There are more than 50 different types of hydroponic systems being used in the world. Although all these systems perform the same functions, the difference is in the arrangement of the physical components of the system. Physiological responses due to nutrients concentration altered the leaf, root and shoot morphology along with affecting chlorophyll content and evapotranspiration rate. However, the study by Kamble *et al*. (2013) suggested that soil tests could also be used to analyse the relationship between each soil nutrient with the pH. Research conducted by Soodan *et al*. (2014) highlighted different analytical techniques that are used for soil analysis including UV-Visible Spectroscopy, Atomic Absorption Spectroscopy and Fourier Transform Infrared Spectroscopy. Previous studies highlighted the fact that increase in concentration of copper in hydroponic nutrient solution or in the soil (added artificially through fertilisers) also affects the growth and various other factors of plants. Therefore, the aim of present study is to investigate the efficiency of hydroponics technique, the morpho-physiological responses of selected vegetable species performed. Moreover, the soil nutrients analysis and hydroponics nutrients uptake analysis were also carried out by using UV-Visible Spectroscopy, Atomic Absorption Spectroscopy and Titration method.

## MATERIALS AND METHOD

### Study Area

The area selected for study was Fatima Jinnah Women University, Rawalpindi, Pakistan located at 33°35’10’’ N; 73°03’54’’ E. The experiments were conducted in spring and summer seasons, i.e., from early May 2023 to end of June 2023 at the greenhouse of the Environmental Sciences Department, Jinnah Women University, Rawalpindi, Pakistan. Five seasonal vegetables were selected to study and contrast the morpho-physiological changes of hydroponic vegetables with those grown in the soil.

### Vegetable Species Selected for the Study

A total of 30 plants (6 from each species) were germinated and used for the experiment. Out of those six, three were used for soil-based experiments and three were used for Hydroponics experiments. Tomato (*Solanum lycopersicum* L.), Eggplant (*Solanum melongena*), Lettuce (*Lactuca sativa*), Green Chili (*Capsicum annuum*) and Okra (*Abelmoschus esculentus*) were the vegetable species selected for the experiment because all these grow efficiently in warmer seasons between temperatures of 25°C–32°C. The experiment was carried out for 60 days. A total of 20 different healthy seeds of each vegetable mentioned above were sown in different germination mediums with the help of a spatula. As for the soil-based experiment, a mixture of compost and soil present in the university’s greenhouse was used. The soil was loamy, sandy and silty. No additional fertilisers were added to the soil till the end of the experiment. Soil was ploughed and levelled before the transfer of seedlings into it. The soil medium plants were irrigated twice daily at 9:00 a.m. and 3:30 a.m.

### Hydroponic Setup

**FIGURE 1:**
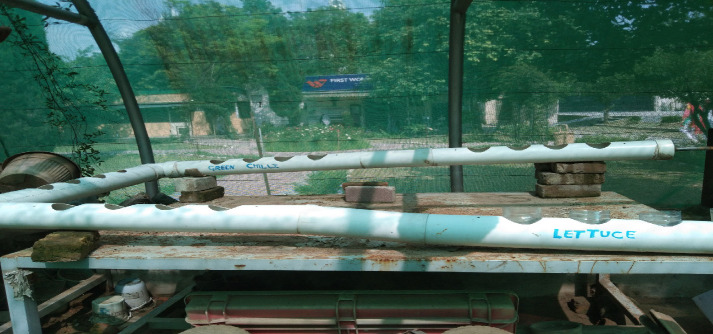
The study’s experimental setup in a greenhouse.

In this experiment, a Deep Water Culture (DWC) hydroponic system was designed in a greenhouse. Instead of distilled/filtered water, we used tap water with a pH between 7.5 and 8. No additional chemical was added to adjust the pH range of the water. As for the loss of water due to evaporation, an additional 10 L to 12 L of water was added to the container before every change (in between periods of every two weeks) without the addition of nutrient solution. No EC (Electrical Conductivity) was maintained during the experiment. The experimental setup consisted of 5 PVC (Poly Vinyl Chloride) pipes, each measuring 5 ft and 4 inches in length, equipped with four holes at regular intervals along the length. These pipes were interconnected to form a “U” shaped structure with a slope. The setup also consisted of one plastic container capable of holding 55 L to 60 L of nutrient-rich hydroponic solution (Fig. 1). A mini water pump was used to circulate nutrient-rich solution from the container to the PVC pipe. The water moved with the slope and drained into the same container, circulating repeatedly. The roots of the selected vegetable were completely exposed to the nutrient-rich solution throughout the study. In an endeavour to minimise environmental impact and optimise resource utilisation, plastic bottles were reused by cutting them in half and adding foam to give support to the plant’s stem. The hydroponic solution was changed every two weeks. Discarded water was mixed with tap water and reused for watering other plants located near the greenhouse to avoid wastage of water (Shrestha & Dunn 2010). The concentrations of all the chemicals used in 100 L of water are given in Table 1.

### Soil Sample Preparation

Wet digestion process was carried out step by step for each soil. Prepared Aqua regia 3:1 HNO_3_ to HCL. A total of 176.25 mL HNO_3_ was added to 56.25 mL HCL for all 15 samples. A total of 11.25 mL HNO_3_ and 3.75 mL HCL were used for each sample. A total of 1 g of soil sample, and the Aqua regia were added into a 250 mL beaker. The beaker was placed over the hotplate at 70°C for 40 min and waited till the solution became transparent. The sample was passed through Whatman Filter paper no. 42. Deionised water was added to the sample to make 50 mL volume in the falcon tube (Maurya *et al*. 2018).

### Water Sample Preparation

In this study, 20 water samples from a hydroponic solution were collected: Two samples at the start of each hydroponic solution (water) changing event and two after a one-week interval. During the experiment, nutrient-rich hydroponic water was changed five times. Samples were just filtered with Whatman Filter Paper no. 42 to remove impurities.

### Nutrients Analysis by UV-Visible Spectroscopy

Copper, Zinc, Manganese, Iron, Magnesium, Calcium and Potassium were analysed using AAS (Atomic Absorption Spectroscopy). Nitrogen, Phosphorus, Sulfur and Boron were analysed using UV-Visible spectroscopy, Chloride by using the Titration method as per previously reported methods. (Mussa *et al*. 2009; Jagessar & Sooknundun 2011; Estefan 2013).

## RESULTS AND DISCUSSION

The results obtained show the different parameters of plants, soil and hydroponic systems that have been investigated with the aim to provide insights on the role these factors, play in promoting significant plant growth and establishing a sustainable hydroponic system.

### Moisture Content and pH of Soil and Water Sample

Findings revealed the percentage moisture content range of 11%–19% in studied soil samples. The average moisture content observed in soil samples was 15.6%. This parameter is responsible for soil microbial and fungal activities. The investigation recorded pH levels within a range of 7.1 to 8.0 for soil samples and 7.0 to 8.0 for water samples.

### Soil and Hydroponic Nutrients Analysis

#### Soil analysis

**FIGURE 2:**
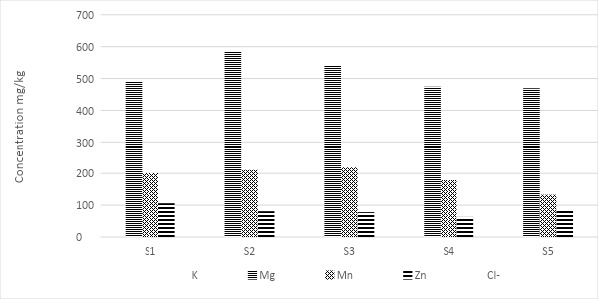

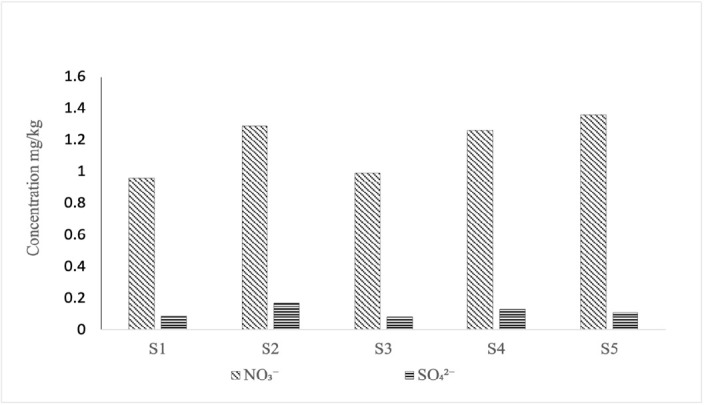

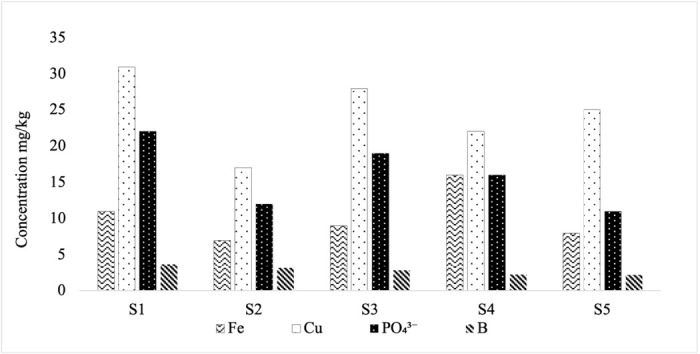

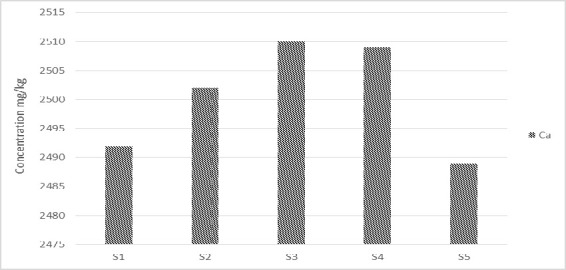
Concentrations of (a) Potassium (K), Magnesium (Mg), Manganese (Mn), Zinc (Zn) and Chloride (Cl−); (b) Nitrates and Sulfates; (c) Iron, Copper, Phosphate and Boron; and (d) Calcium, measured in soil samples.

The study involved the analysis of concentrations of different anions and cations in soil samples using various methods and analytical techniques. Concentrations of these elements individually affect the growth and development of plants. For experimentation, five different soil samples denoted as S1, S2, S3, S4 and S5 were used to cultivate soil-based plants, namely Tomato, Green Chili, Okra, Lettuce and Eggplant, respectively. The average concentrations of Potassium (K), Calcium (Ca), Magnesium (Mg), Iron (Fe), Manganese (Mn), Zinc (Zn), Copper (Cu), Chloride (Cl), Nitrate (NO_3_^−^), Phosphate (PO_4_^3−^), Sulfate (SO_4_^−2^) and Boron (B) observed in soil samples were 147 mg/kg, 2,500 mg/kg, 470 mg/kg, 10 mg/kg, 188 mg/kg, 83 mg/kg, 25 mg/kg, 87 mg/kg, 1.7 mg/kg, 0.11 mg/kg, 16 mg/kg and 2.8 mg/kg, respectively, as shown in Fig. 2. The concentration of K, Mg, Mn, Zn and Cl− measured in soil samples (Fig. 2a). The concentration of nitrates and sulfates measured in soil samples shown in Fig. 2b and concentration of Fe, Cu, PO_4_^3−^ and B measured in soil samples (Fig. 2c), and the concentration of Ca measured in soil samples is represented in Fig. 2d.

#### Water analysis

Concentration of following anions and cations in the hydroponic water samples was analysed using different methods and analytical techniques. These cations and anions were supplied to the plants using the different chemicals mentioned. Concentrations of these elements individually affect the growth and development of plants. Excessive or deficient supply of any of these elements causes several diseases in plants and sometimes causes death to plants and soil microbes. Samples W1, W3, W5, W7 and W9 were taken immediately after mixing the nutrient-rich solution in the water for hydroponic plants, whereas W2, W4, W6, W8 and W10 were taken after an interval of 1 week after every change. Results showed the percentage of each anion and cation taken up by the plants every week. The average concentrations of K, Ca, Mg, Fe, Mn, Zn, Cu, Cl^−^, B, NO_3_^−^, SO_4_^−2^ and PO_4_^3−^ observed in water samples taken during the start of every change of hydroponic water were 323 mg/L, 203 mg/L, 165 mg/L, 3.702 mg/L, 0.84 mg/L, 3.042 mg/L, 0.43 mg/L, 118 mg/L, 0.138 mg/L, 140 mg/L, 163 mg/L and 89 mg/L, respectively. Whereas, the average concentration of these cations and anions observed in water samples taken after every 1 week of change of hydroponic water was 166 mg/L, 84 mg/L, 62 mg/L, 0.78 mg/L, 0.46 mg/L, 0.65 mg/L, 0.21 mg/L, 68 mg/L, 0.05 mg/L, 74 mg/L, 66 mg/L and 44 mg/L. In our results, 52%, 58%, 62%, 79%, 45%, 79%, 51%, 42%, 64%, 47%, 60% and 51% of K, Ca, Mg, Fe, Mn, Zn, Cu, Cl^−^, B, NO_3_^−^, SO_4_^−2^ and PO_4_^3−^, respectively had been taken up by the plants during the 60 days experiment.

### Growth and Germination

After 20 days of seed sowing, the germination percentage of all five plant species was recorded separately. The 19 seeds were successfully germinated for Tomato and Eggplant, resulting in a splendid germination percentage of 95%. Both Green Chili and Okra showed impressive germination percentages of 100%, which means all the seeds were successfully germinated. However, Lettuce showed a minimum percentage of 85%.

### Plant Length

Seedlings used for both hydroponics and soil-based experiments were almost similar in length. The length of the Tomato seedlings was between 14 cm and 15 cm. The length of the Eggplant seedlings was between 8 cm and 9 cm. The length of the Lettuce seedlings was 7 cm to 10 cm. The length of the Green Chili seedlings was 8 cm to 9 cm, and the length of the Okra seedlings was 10 cm to 14 cm.

#### Soil plants

After 60 days, soil-grown tomato plants reached 28 cm–29 cm. Eggplants grew to 15 cm–18 cm, and lettuce achieved 14–16 cm. Among green chili plants, G1 showed retarded growth at 15 cm, whereas the others reached 20 cm and 30 cm. Okra plants exhibited a final length of 25–27 cm.

#### Hydroponic plants

In hydroponics, tomato plants reached 31 cm–32 cm, eggplant 24 cm–26 cm, and lettuce exhibited a uniform length of 16 cm. The green chili plant G1 displayed exceptional growth at 45 cm, while the other two plants measured 35 cm and 28 cm. Okra plants reached 31 cm–32 cm.

### Length of Roots

Plant root measurements were done with a measuring scale in cm twice during the experiment: once when seedlings were transferred to their respective medium and again after 60 days of seedling transplantation.

#### Soil plants

Tomato roots ranged from 7 cm to 8 cm. Eggplant roots reached 6 cm–8 cm, lettuce 6 cm–7 cm, and okra 5 cm–7 cm. Green chili G1 showed retarded root growth at 4 cm, whereas other plants measured 6 cm–7 cm.

#### Hydroponic plants

Tomato roots reached 8 cm–9 cm, eggplant 7 cm–8 cm, and lettuce 7.5 cm–10 cm. Green chili roots ranged from 8 cm to 9 cm, while okra measured 6.5 to 8 cm.

### Total Number of Leaves per Plant Species

Seedlings utilised in both hydroponics and soil-based experimental setups had almost a similar number of leaves on their stems. The leaves counted manually for all these seedlings were in between a range of 3 to 5.

#### Soil plants

Tomato plants developed 11–13 leaves, eggplant 5–7 leaves, lettuce 4–6 leaves and okra 9–11 leaves. Green chili plants had 11, 14 and 16 leaves for G1, G2 and G3, respectively.

#### Hydroponic plants

Tomato plants showed variation, with T1 at 12 leaves, T2 at 16 leaves and T3 at 22 leaves. Eggplants developed 7–8 leaves, lettuce 6–7 leaves and okra 11–12 leaves. Green chili plants achieved 19, 22 and 17 leaves for G1, G2 and G3, respectively.

### Weight of Plants

The seedlings of all the plants used in both soil-based and hydroponics experimental setup showed almost the same weight in grams which was ranging between 3 g to 5 g.

#### Soil plants

Tomato plants weighed 16 g–17 g, eggplant 13 g–14 g, lettuce 18 g–19 g, and okra 22 g–26 g. Green chilli G1 had the lowest weight at 11 g, while G2 and G3 weighed 18 g and 22 g, respectively.

#### Hydroponic plants

Tomato plants weighed 20 g to 22 g, eggplant 22 g to 23 g, and lettuce 18 g to 19 g. Green chili plants weighed 27 g to 39 g, and okra ranged from 26 g to 31 g.

Overall, hydroponic cultivation consistently enhanced plant growth compared to soil-based systems. Plant height, root length, leaf production and biomass were generally higher under hydroponic conditions. Tomato and okra showed moderate increases in length and weight, while eggplant and green chili exhibited more pronounced improvements, particularly in previously slower-growing individuals like green chili G1. Root systems were more robust in hydroponics, supporting better nutrient uptake and overall development. Leaf number was also higher in hydroponic plants, reflecting more vigorous vegetative growth. These results indicate that hydroponic systems provide a more favourable environment for growth, likely due to optimised nutrient availability and controlled water supply, leading to improved overall plant performance compared to traditional soil cultivation.

### Evapotranspiration Rate (E) in Plant

Plant efficiency analyser LCI T instrument was used to observe the evapotranspiration rate of plants. For soil-based plants, the maximum average recorded evapotranspiration rate was 0.27 mg/m^2^/s in Lettuce, whereas the minimum average recorded value was 0.10 mg/m^2^/s for tomatoes. In hydroponic plants, the maximum average evapotranspiration rate was 0.27 mg/m^2^/s for green chili and the minimum recorded average value was 0.11 mg/m^2^/s for tomatoes.

### Carbon in Intercellular Spaces (Ci) of Plants

**FIGURE 3:**
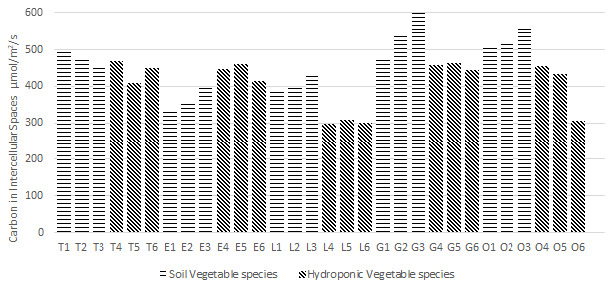
Average Carbon in Intercellular Spaces (Ci) measured for each studied Vegetable species.

Plant efficiency analyser LCI T instrument was used to observe the carbon in intercellular spaces (Ci) of plants. For soil-based plants, the maximum average recorded Ci rate was 865 μmol/m^2^/s in Okra whereas the minimum average recorded value was 387 μmol/m^2^/s for tomatoes. In hydroponic plants, the maximum average Ci rate was 770 μmol/m^2^/s for green chili and the minimum recorded average value was 298 μmol/m^2^/s for tomatoes are shown in Fig. 3.

### Photosynthetic Rate (A) of Plants

**FIGURE 4:**
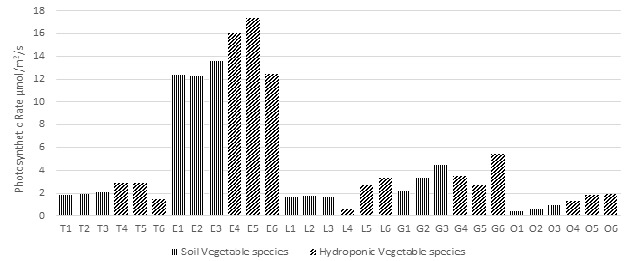
Average photosynthetic rate (A) measured for each studied Vegetable species.

Plant efficiency analyser LCI T instrument was used to observe the photosynthetic rate of plants. For soil-based plants, the maximum average recorded photosynthetic rate was 13.6 μmol/m^2^/s in Eggplant, whereas the minimum average recorded value was 0.46 μmol/m^2^/s for Okra. In hydroponic plants, the maximum average photosynthetic rate was 17.4 μmol/m^2^/s for Eggplant and the minimum recorded average value was 0.7 μmol/m^2^/s for Lettuce, as shown in Fig. 4.

### Stomatal Conductance (gs) in Plants

**FIGURE 5:**
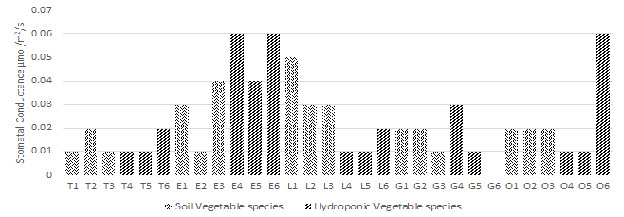
Average stomatal conductance rate (gs) measured for each studied Vegetable species.

Plant efficiency analyser LCI T instrument was used to observe the stomatal conductance rate of plants. For soil-based plants, the minimum average recorded stomatal conductance rate was 0.01 μmol/m^2^/s in Green Chili, Tomato and Eggplant, whereas the maximum average recorded value was 0.05 μmol/m^2^/s for Lettuce. In hydroponic plants, the minimum average stomatal conductance rate was 0.00 μmol/m^2^/s for Green Chili and the maximum recorded average value was 0.06 μmol/m^2^/s for Okra and Eggplant, as shown in Fig. 5.

The average pH value in the water and soil samples was determined to be 7.6, indicating a medium alkaline condition. This finding is consistent with Rajput *et al*.’s (2017) study analysing the physico-chemical characteristics of guava orchards in Larkana, Pakistan. Soil pH is pivotal for promoting plant growth, as extreme acidic or alkaline pH levels can adversely impact microbial growth and soil structure, ultimately leading to inhibited plant growth (Cho *et al*. 2016). Studies suggest that the optimal soil pH range for plant growth lies between 5.5 and 6.5, mirroring the pH value recorded for the hydroponic system (Singh & Dunn 2016). However, hydroponic plant species also exhibit growth constraints in low pH conditions below 5.7 (Anukool *et al*. 2012). Our potassium (K) concentration results align with Barbagelata’s study (2006), which reported K concentrations ranging from 140 mg/kg to 210 mg/kg in soil samples. Schulte’s (1992) research highlights that neutral soils typically exhibit high pH and magnesium (Mg) concentrations exceeding 500 mg/kg. On a global scale, Mn content varies from 270 ppm to 525 ppm (Kabata-Pendias 2000). However, the zinc (Zn) content in our soil samples exceeded the permissible limit set by the World Health Organization (WHO) at 50 mg/kg (Ogundele *et al*. 2015). The normal chloride (Cl−) concentration in soils is approximately 100 mg/kg (Geilfus 2019), falling within the global range of 56 ppm to 305 ppm (Kabata-Pendias 2000).

Excessive nitrate uptake through various food sources can lead to gastrointestinal cancer (Rao & Puttanna 2000). Our results indicate an extremely low nitrate concentration, necessitating supplementation through fertilisers. Our copper (Cu) content aligns with the WHO permissible limit of 36 mg/kg (Ogundele *et al*. 2015). Soil boron content worldwide ranges from 2 mg/kg to 200 mg/kg (Arunkumar *et al*. 2018). Comparable to our results, research notes that clayey soils tend to have calcium concentrations exceeding 2500 mg/kg (Espinoza *et al*. 2012).

Research by Chen *et al*. (2021) demonstrates that varying phosphorus concentrations (1 mM–5 mM) supplied as KH_2_PO_4_ do not impact leaf number or plant height. Our lettuce results agree with de Souza *et al*.’s (2019) study, which showed hydroponic lettuce reaching 33 cm in length, twice that of our hydroponic lettuce, while soil-based lettuce achieved 15 cm, like ours. Olutola *et al*.’s (2020) comparative study on okra growth in hydroponics and conventional farming aligned with our hydroponic okra height of 38 cm and their conventional farming height of 31 cm. Ahmadi and Souri’s (2020) four-month chili pepper study yielded an 88 cm height under controlled conditions. Given our G4 hydroponic plant’s growth, extending the experiment to 4 months could have achieved similar heights. Overall, hydroponic system-grown plants exhibited longer roots than their soil-based counterparts. Siddiq (2012) found that Tomato root length was maximised with higher applied CaC_2_ (15 mg). Grunhofer *et al*.’s (2021) hydroponic P. × canescens roots reached 13 cm under controlled conditions, surpassing our experiment’s roots. Optimal root, shoot and leaf growth rates occur at a flow rate of 4 L/min (Baiyin *et al*. 2021). Excessive iron (Fe) levels (up to 400 ppm) hinder root growth (Turhadi *et al*. 2018). For lettuce, de Souza *et al*. (2019) reported 50 cm hydroponic and 30 cm soil lettuce roots, significantly more than our results. In terms of leaf numbers, our hydroponic-grown plants exceeded their soil-based counterparts. Lettuce leaves have economic importance in salads and fast foods (Lipton & Ryder 2021). Okra leaves contain silica, protecting against harmful UV rays (Pierantoni *et al*. 2017). Changes in auxin levels lead to diverse tomato leaf forms (Shwartz et al. 2016). A four-month chili pepper study (Ahmadi & Souri 2020) recorded 94 leaves. Extending our experiment to 4 months could yield comparable numbers for G2 (soil) and G4 (hydroponic). Olutola *et al*. (2020) in a 7-month okra study reported 116 hydroponic and 81 conventional farming leaves, potentially achievable in our experiment with an extended timeframe.

## CONCLUSIONS AND RECOMMENDATIONS

The temperature observed during the course of the experiment was between 30^o^C–37^o^C that was best for the growth of selected vegetables. All selected seeds showed an impressive germination rate. Different morpho-physiological parameters of selected plant species were studied in soil and hydroponic experiments. However, Green Chili showed the highest growth in terms of height, weight and number of leaves. However, Lettuce showed the lowest growth rate in both experiments. The overall result showed that the hydroponic system had provided positive results for the Selected Vegetable as compared to soil-based plants. The experiment revealed no plant mortality, suggesting optimal growth conditions. However, soil analysis was carried out to identify the concentration of different anions and cations. The concentrations of all the elements in the soil were normal (neither toxic nor deficient), and no additional fertilisers were added to the soil. Water analysis results showed percentages of different anions and cations taken up by all the plants during the experiment. No EC was calculated for soil and water samples. This research results showed that the hydroponic system has the ability to achieve related sustainable development goals such as climate action, responsible consumption and production, industry, innovation and infrastructure, good-health and wellbeing, and zero hunger. However, the duration of experiment is recommended to increase and wait for the fruit development. The fruit nutrition testing comparison of hydroponic and soil-grown plants should be carried out to understand and ensure which system is primarily promoting human and environmental health safety. The study should be extended to make a nutrient-rich hydroponic solution using a smaller number of chemicals. It is recommended that formulation of hydroponic solution incorporates Epsom salt and NPK fertiliser or natural compost. The hydroponic technique should be used in an open environment under uncontrolled conditions. In addition to current plant selection, experiments should be conducted on climbing vegetables and fruits too. To promote resource efficiency, usage of artificial electricity sources for providing continuous flow of hydroponic water to the roots of plants should be replaced with renewable solar energy.

## Figures and Tables

**TABLE 1:** Particle size distribution of AgNPs2.

Solution “A” chemicals	%	Solution “B” chemicals	%
Calcium Nitrate	26.6	Iron Chloride	0.27
Magnesium Sulfate	26.6	Manganese Sulfate	0.05
Mono Ammonium Phosphate	5.3	Zinc Chloride	0.05
Mono Potassium Phosphate	6.39	Copper Sulfate	0.03
Potassium Nitrate	7.99	Boric Acid	0.03
Potassium Sulfate	26.6	Sodium Molybdate	0.005

(Source: Mattson 2018)
